# Rift Valley Fever Virus, Japanese Encephalitis Virus, and African Swine Fever Virus: Three Transboundary, Vector-Borne, Veterinary Biothreats With Diverse Surveillance, and Response Capacity Needs

**DOI:** 10.3389/fvets.2019.00458

**Published:** 2019-12-13

**Authors:** Rebekah C. Kading, Edward O. Abworo, Gabriel L. Hamer

**Affiliations:** ^1^Arthropod-Borne Infectious Disease Laboratory, Department of Microbiology, Immunology and Pathology, Colorado State University, Fort Collins, CO, United States; ^2^International Livestock Research Institute, Nairobi, Kenya; ^3^Department of Entomology, College of Agriculture and Life Sciences, Texas A&M University, College Station, TX, United States

**Keywords:** emerging arboviruses, ticks, mosquitoes, biosurveillance, introduction, guidelines

## Abstract

Early detection of emerging foreign animal diseases is critical to pathogen surveillance and control programs. Rift valley fever virus (RVFV), Japanese encephalitis virus (JEV), and African swine fever virus (ASFV) represent three taxonomically and ecologically diverse vector-borne viruses with the potential to be introduced to the United States. To promote preparedness for such an event, we reviewed the current surveillance strategies and diagnostic tools in practice around the world for these emerging viruses, and summarized key points pertaining to the availability of existing guidelines and strategic approaches for early detection, surveillance, and disease management activities. We compare and contrast the surveillance and management approaches of these three diverse agents of disease as case studies to emphasize the importance of the ecological context and biology of vectors and vertebrate hosts. The information presented in this review will inform stakeholders of the current state of surveillance approaches against these transboundary foreign animal disease which threaten the United States.

## Introduction

Rift Valley fever virus (RVFV), African swine fever virus (ASFV), and Japanese encephalitis virus (JEV), are three vector-borne veterinary pathogens with the potential to invade the United States (US). All of these viruses are notifiable animal diseases (1), and Rift Valley fever (RVF) is also included on the “World Health Organization (WHO) Blueprint of priority pathogens” (2). While these viruses would all have devastating health and economic impacts, the ecological context and potential transmission dynamics of each are quite different and would require unique preparedness and mitigation efforts. All of these agents are capable of vector-borne as well as direct transmission (Table 1). JEV and RVFV are also zoonotic and capable of causing disease in humans, and the potential vector, domestic, and wild animal species involved in enzootic and spillover transmission are variable. Routes of potential entry into the US span importation of infected vectors (all three agents), viremic travelers (RVFV), infected animal products (ASFV), or contaminated fomites (ASFV), hence requiring very different preparedness efforts. This complexity in disease ecology and introduction potential presents a challenge to our health infrastructure to prepare for one or multiple such events.

**Table 1:** Similarities and differences among the invasion and establishment of Rift Valley fever virus, Japanese encephalitis virus, and African swine fever virus into the United States.

	**Rift Valley fever**	**Japanese encephalitis**	**African swine fever**
Human pathogen	Yes	Yes	No
Vaccine available	No^*^	Yes	No
Vector-borne potential	Mosquitoes	Mosquitoes	Ticks
Direct transmission	Yes	Yes	Yes
Likely wildlife reservoir	Deer	Birds	Feral hogs
Affected domestic hosts	Goats, sheep, cattle	Pigs	Pigs
Possible route of entry	People, mosquitoes	Mosquitoes	Swine products, fomites

* *Currently there is no vaccine licensed in the U.S., but multiple vaccines have or are being developed*.

The risk of introduction and establishment for RVFV, JEV, and ASFV to the US have all been investigated. The emergence of RVFV on the Arabian Peninsula (3) and recent reports of RVFV-infected travelers returning to France and China from areas of active RVFV transmission (4, 5) demonstrate the feasibility of pathogen importation via air travel. Considering five potential pathways of entry, Golnar et al. predicted the most likely introduction would be through an infected human traveler, but invasion of infected mosquitoes by plane or ship also carried quantifiable risk (6). In a separate qualitative analysis, Kasari et al. also concluded that RVFV could feasibly enter the US through several pathways including importation of infected animals, people, vectors, or live virus (7). If introduced, many competent mosquito vectors exist in the US (8, 9), and maintenance of the virus in mosquito populations by vertical transmission is possible (10–12). Deer have recently been shown to be a susceptible amplifying host (13) with viremias capable of infecting blood-feeding mosquitoes, which significantly increases the probability of enzootic establishment (14). Intensive livestock production in the US also creates a vulnerable environment for virus transmission among human and livestock communities by opportunistic mosquito vectors adapted to agricultural environments (15). Barker et al. modeled the potential for RVFV transmission in California considering local vectors, hosts and environmental conditions, but understanding the long-term persistence of RVFV depended on the parameterization of the models with data that were not available at the time this model was developed (16).

Japanese encephalitis virus strains have a history of invasion and establishment among new areas in Asia. Molecular epidemiological evidence suggests that JEV strains are introduced from continental south-east Asia to Japan regularly (17–19). Introductions of JEV within Asia and between Asia and Australia are believed to be through either infected windblown mosquitoes or birds, where the virus then circulates enzootically among native birds and *Culex* spp. mosquitoes (20–22). Considering seven possible pathways of JEV entry into the US, entry of infected adult mosquitoes through air travel was the most likely scenario, however, the risk of establishment of JEV in the US following this type of introduction was variable (23). Several parameters complicated the establishment in the US, including a relatively short host viremia, unpredictable contact rates between hosts and vectors, particularly near airports, virus strain and genotype differences, and the cross-protection of JEV with other flaviviruses endemic to the US (23). North American *Culex* mosquitoes have been shown to be competent for infection with JEV under experimental conditions (24, 25). The predictable and stochastic factors influencing an introduction are complex, which makes risk assessment efforts challenging.

African swine fever virus has demonstrated its ability to cross national borders and establish in diverse geographical areas around the globe (26–29). A unique risk for introduction of ASFV is that this DNA virus is exceptionally stable in the environment, and remains viable in animal products. This property opens a potential route of entry not available to RVFV and JEV. If ASFV invades the US, competent vertebrate amplifying hosts as well as transmission-competent soft ticks are known to be present, although many questions remain (30). California, Texas, and Florida were among the states with the highest predicted risk for ASFV introduction through imported swine or swine products (31, 32). Further, these states plus much of the southwestern US were predicted to have increased risk for ASFV establishment should this virus be introduced. Additionally, operational procedures that characterize small-scale and organic pig producers throughout the southern US made these enterprises particularly vulnerable to ASFV circulation given the potential exposure to feral swine or *Ornithodoros* spp. soft ticks (32).

Preventing establishment of any or all of these viral pathogens would require biosecurity and health infrastructure to have a broad focus able to evaluate the risk of introduction and establishment of these disease agents. For example, monitoring passengers with febrile illness entering via airplane from RVFV-endemic countries, inspecting aircraft entering the US from JEV and RVFV-endemic countries for mosquitoes, testing swine products for ASFV, educating agricultural, wildlife, and veterinary professionals to recognize signs and symptoms of infection with any of these agents, conducting surveillance on a diversity of wildlife species spanning birds, deer, and feral swine, and being prepared to mitigate local vector-borne transmission through mosquito and tick vector control. As an example of extreme caution, the 2019 World Pork Expo to be held in June in Des Moines, Iowa was canceled by the National Pork Producers Council due to the concern for the risk of ASFV infected products or fomites being introduced from infected countries (33). Executing the scope and complexity of these activities effectively with limited resources will make identification and early response to any of these agents particularly challenging. Diagnostic labs in the US testing samples from animals or arthropod vectors do not routinely test for these three agents specifically. Still, an extensive national network of veterinary diagnostic laboratories, and valuable knowledge, guidelines, and diagnostic tools exist for each of these viral pathogens to help prepare for such events. The following sections compile information on the current status of surveillance practices and diagnostic tools in use for RVFV, JEV, and ASFV.

## Rift Valley Fever Virus

Rift Valley fever is a notifiable disease to the World Organization for Animal Health (OIE) and is additionally cross-listed as a select agent with the USDA and US Department of Health and Human Services (HHS). Due to the RVF disease severity, sizeable outbreaks, and the significant health and economic impacts, national and international guidelines for RVF surveillance and response activities have been developed.

### Surveillance

The ability to predict likely times when an outbreak may occur would provide incredible power toward the timely mobilization of intervention and control measures. For RVF outbreaks in East Africa, a close association between epizootic activity and the occurrence of El Niño has been documented (34), providing an opportunity for monitoring weather patterns as an early indicator of conditions consistent with historic outbreak conditions. In particular, NASA has developed an outbreak risk mapping system for RVFV that inputs a variety of satellite measurements to produce a dynamic map of areas at potential risk of virus transmission. This “*Rift Valley Fever Monitor*” (35), records monthly RVF risk for Africa and the Middle East based on interpretation of satellite vegetation indices, and is maintained by the USDA and NASA (11, 36). In 2008, the WHO convened a joint meeting of experts with the United Nations Food and Agriculture Organization (FAO) to share experiences and discuss the predictability of forecasting models for RVF, and develop guidelines for improving prediction and response capabilities to RVF outbreaks (37). How these predictive models might translate to environmental risk in the US has been approached (16) but needs further study. Still, the precedent has been set for a potential contribution of climate monitoring and predictive modeling into RVFV outbreak prevention.

In 2014, the African Union-InterAfrican Bureau for Animal Resources (AU-IBAR) published a framework to support consistency and coordination of disease control activities, referred to as the “Standard Methods and Procedures (SMPs) for control of RVF in the Greater Horn of Africa” (38). The overall approach concerning the development of these SMPs relies on the coordination of disease prevention and control activities in each participating region (i.e., Greater Horn of Africa) to a set of regional minimum standards and procedures that align with the OIE standards (38). This SMP document provides valuable information on surveillance and epidemiology, diagnosis and laboratory detection, disease control, disease reporting and information management, RVF and trade, and risk analysis and risk mapping, and is supported by specific Standard Operating Procedures (SOPs) for each subject area in accordance with the structure and capabilities of each nation.

Of particular relevance to a potential introduction of RVFV to the US, the AU-IBAR SMP document lists recommendations for surveillance approaches in areas which have not yet reported RVFV activity, in order to promote early detection and rapid response to a virus introduction. Among these recommendations are a combination of passive (continuous) and active surveillance (as needed, according to perceived risk) (38) (Table 2). Passive surveillance would involve the distribution of educational materials to veterinary personnel, and syndromic surveillance conducted by veterinarians, livestock handlers, and wildlife agencies. A specific reporting network is outlined for when suspicious cases are identified. Active surveillance would include serosurveillance, syndromic surveillance, and the regular monitoring of RVFV antibody levels in sentinel herds or flocks. Enhanced vector surveillance is recommended during interepidemic activities, to monitor for increased virus infection rates in mosquito populations. Supporting these surveillance activities should be administrative preparations, such as capacity building and training, and development of a policy framework to support surveillance activities (38).

**Table 2:** Guidelines and control recommendations for Rift Valley fever virus, Japanese encephalitis virus, and African swine fever virus.

**Pathogen**	**Available Guidelines and Resources**	**Disease control recommendations**	**References**
Rift Valley Fever virus	Standard Methods and Procedures (SMPs) for control of Rift Valley Fever (RVF) in the Greater Horn of Africa	Mass vaccination—Smithburn vaccine or Clone 13 Vector control Animal quarantine and movement control Public education	(38)
	Rift Valley fever surveillance: FAO Animal Health and Production Manual No. 21	Mass vaccination Vector control Communication and public outreach	(39)
	The Foreign Animal Disease Preparedness and Response Plan (FAD PreP)—Disease Response Strategy: Rift Valley Fever	Animal quarantine and movement control (state level) Possible restrictions on interstate commerce Vaccination, if vaccine is available Possible euthanasia and mass depopulation of affected animals Wildlife management Vector control	(40)
Japanese Encephalitis Virus	“Japanese encephalitis virus infection, diagnosis and control in domestic animals”	Vaccination of animals Vector control	(41)
	WHO-recommended standards for surveillance of selected vaccine-preventable diseases	Vaccination of people, Assessment of the impact of vaccination Syndromic surveillance for acute encephalitis syndrome Standardized, aggregate, data reporting	(42)
	Manual for the Laboratory Diagnosis of Japanese Encephalitis Virus Infection	Detailed specimen handling, laboratory testing and data management recommendations	(43)
	The Foreign Animal Disease Preparedness and Response Plan (FAD PReP)—Disease Response Strategy: Japanese encephalitis virus	Quarantine and movement control Stamping out—swine depopulation on infected premises within 24 h Vaccination, if vaccine is available Public outreach campaigns Wildlife management Vector control	(40)
African Swine Fever Virus	African swine fever. In OIE terrestrial manual 2012.	Detailed information provided on specific diagnostic procedures	(44)
	The Foreign Animal Disease Preparedness and Response Plan (FAD PReP)—Disease Response Strategy: Rift Valley Fever (2013)	Biocontainment and stamping out on infected premises Public outreach campaigns Prevent contact between feral and commercial swine Possible feral swine depopulation in affected areas Quarantine and movement control, particularly for fomites Possible complete movement standstill for live swine	(45)
	USDA: Swine Hemorrhagic Fevers: African and Classical Swine Fever Integrated Surveillance Plan	Detailed sampling and testing recommendations, case definitions, and reporting for commercial, backyard, and feral swine	(46)

The rapid deployment of available vaccines may aid considerably in curtailing virus circulation. RVF vaccines currently being deployed under emergency circumstances in Africa include the formalin-inactivated attenuated Smithburn strain. The Smithburn strain vaccine is “recommended for use in pregnant animals and in RVF-free countries experiencing outbreaks” (47); even though multiple boosters may be required. At the time of this writing, there is no RVF vaccine approved for human or veterinary use in the US, however RVF vaccine research and development is being pursued (48–51). If the decision to vaccinate is made, the vaccine must meet the standards in the “OIE Manual of Standards for Diagnostic Tests and Vaccines.” The final decision to vaccinate will be made by the US Department of Agriculture (USDA) Animal Plant Health Inspection service (APHIS) VS Deputy Administrator (US Chief Veterinary Officer) (40). Once vaccination is implemented, the ability to differentiate among infected and vaccinated animals (DIVA) will be essential in order to assess vaccine coverage as compared with virus exposure levels (48, 52).

The USDA APHIS has published “The Foreign Animal Disease Preparedness and Response Plan (FAD PReP)—Disease Response Strategy: Rift Valley Fever” (2013) (40) (Table 2). This document is intended to provide responders with the critical information necessary to mount an effective response effort against RVF in domestic livestock in the US (40). This document recommends approaches to disease control and eradication in the event of an introduction, including the roles and responsibilities of US government agencies (40). These control and eradication strategies are based on four epidemiological principles: “1. Prevent contact between RVFV and susceptible animals (i.e., quarantine and movement control), 2. Stop the production of RVFV by infected or exposed animals, 3. Stop the production of RVFV by insect vectors (i.e., vector surveillance and control), and 4. Increase the disease resistance of susceptible animals to RVFV (i.e., vaccination)” (40). Vector surveillance is only considered in the context of an outbreak and rapid response situation in these guidelines, and not as a potential strategy for early detection. Because of the diverse infrastructure for how vector surveillance and control is managed, these decisions would be left to the agency responsible for conducting vector surveillance activities at the county or municipal government level (40). While these guidelines take some important first steps in providing a framework for priority activities by responsible parties, additional planning is needed to determine mechanisms for inter-agency collaboration during surveillance and response efforts, and identify capacity gaps for implementation of this strategic plan on the ground in an emergency situation. Further, no plan is outlined for conducting pre-introduction surveillance activities, either active or passive, as is detailed in the USAID plan (38). These gaps should be addressed for diseases, such as RVFV which are considered serious risks for introduction.

In December of 2006, the “APHIS Centers for Epidemiology and Animal Health in Fort Collins, Colorado,” hosted a working group meeting comprised of representatives from several academic institutions and government agencies to discuss the state of research and preparedness for RVF (53). Participants presented and discussed key research areas to improve the national capacity to respond to an introduction of RVFV, including more research regarding the relative importance of North American mosquitoes to transmission of RVFV, and the role of wildlife species to enzootic maintenance of RVFV circulation. Since the time of this publication, additional research has been conducted on the vector competence of North American mosquito vectors for RVFV (8, 9, 15), and the susceptibility of deer (13). Discussion was held on interagency cooperation and collaboration during outbreak events. On the response side, organized vector control in the US is principally concentrated in larger cities with high human densities. Rural landscapes where the enzootic cycle of RVFV is likely to amplify and spill-over into human and animals generally lacks organized vector control. Vector control in large rural landscapes often relies on partnerships with military capability (54). Similarly, there is a deficit in veterinary surveillance, and the tools to perform this type of surveillance safely. Veterinary surveillance is and will be very important for early detection of invasive vectors and pathogens. As an example, observations of Myiasis in Florida key deer populations in 2016 led to the discovery of an invasion of the screw worm fly, *Cochliomyia hominivorax* (Coquerel), which had been eradicated from the US since 1959 (55). Re-eradication of this fly was declared in March of 2017, after 35 releases of sterile flies (55). Participants of the RVF Working Group recommended training relevant professionals “to recognize the early signs of an RVF epizootic or epidemic,” and identified a shortage in high containment biosafety laboratories to safely conduct the necessary research (53).

### Diagnostic Tools

The diagnostic tests for identification of RVFV listed in the “OIE Manual of Diagnostic Tests and Vaccines for Terrestrial Animals (2012)” include virus isolation, reverse transcriptase polymerase chain reaction (RT-PCR)-based assays, an agar gel immunodiffusion (AGID) assay, an enzyme-linked immunosorbant assay (ELISA), and immunohistochemistry (40, 44). Mansfield et al. comprehensively reviewed clinical diagnostic methods for RVFV infection as well as advancements in vaccine development (56). Therefore, only some representative assays for these widely-used techniques are highlighted below:

#### Serological Diagnosis

VanVuren and Paweska developed an ELISA based on the capture of the nucleocapsid protein (NP) (57). Alternatively, a monoclonal antibody-based competitive ELISA developed by Kim et al. for the detected antibodies against RVFV in goats and cattle with a sensitivity/specificity of 94.7/99.7% between 9 and 11 days post-inoculation (58). A multiplex fluorescence microsphere immunoassay (FMIA) also detected cattle and sheep IgM and IgG antibodies to the RVFV glycoprotein, non-structural proteins, and nucleoprotein (59). They also reported that the “N protein was the best target for early detection of infection” (59). A novel lateral flow strip test has recently been developed based on detection of the N protein, for use as a pen side diagnostic (60). Importantly, McElroy et al. developed an ELISA that can be used to differentiate infected from those that had been vaccinated a ΔNSm/ΔNSs double-mutant vaccine (48, 52).

The MAGPIX® and the Luminex xMAP platforms have also been used to simultaneously detect IgG antibodies against a multitude of key emerging viral species and families including RVFV (61, 62). Therefore, many options exist for pathogen detection, depending on the goals of the surveillance program and institutional capabilities.

#### Molecular Detection

##### qRT-PCR

Bird et al. developed a robust pan-RVF virus quantitative RT-PCR assay (63). Primers and a probe were designed from the genome alignments of 40 virus isolates derived from representative vector and vertebrate hosts over time. This assay was successfully validated on human RVF clinical samples, and has clinical diagnostic capabilities out to at least 10 days post-symptom-onset. The assay developed by Wilson et al. amplifies the L and M segments as confirmatory targets, and includes a third target gene, NSs, which is deleted in multiple vaccine candidates (64). This approach will be tremendously advantageous for simultaneously monitoring vaccine coverage vs. virus spread.

#### Isothermal Amplification

Recently, isothermal nucleic acid amplification methods have gained popularity for their ability to more rapidly detect pathogen nucleic acids directly in a clinical or field sample, and not necessarily require RNA extraction. Eular et al. developed an “isothermal ‘recombinase polymerase amplification (RPA)' assay” on an “ESEquant tubescanner device.” While lacking in sensitivity compared to qRT-PCR, the portability of this system could be a potential tool for field, pen side or bedside diagnostics (65).

#### Nanotrap Particles

One innovative new approach to sample preparation, which also addresses sensitivity issues that might arise during surveillance, is the use of nanotrap particles to improve the detection of RVFV in low-titer samples by ~100-fold (66). Inactivation of RVFV by detergents or heat treatments did not affect the efficacy of virus binding to nanotrap particles, demonstrating that field samples can be safely inactivated and processed with nanotrap enrichment method to maximize sensitivity and maintain safety precautions (66).

#### Multiplex Pathogen Detection

Rather than focus on specific detection of RVFV, there are several multiplex assays that incorporate RVFV into the testing panel. Fajfr et al. developed multiplex assays targeting hemorrhagic filoviruses, arenaviruses, and bunyaviruses including RVFV, using previously-published primers (67). These assays are optimized for several types of real-time PCR instruments, including a portable “Ruggedized Advanced Pathogen Identification Device (R.A.P.I.D.)” (Idaho Technology, Inc.). Liu et al. developed a real-time PCR-based TaqMan array card (TAC) that can screen up to eight samples within 3 h for up to 26 agents simultaneously (68). The comprehensive panel includes 15 viruses, eight bacteria, and three protozoa (68). Another multiplex platform is the Lawrence Livermore Microbial Detection Array which “detects 10,261 species of microbes including 4,219 viruses, 5,367 bacteria, 293 archaebacteria, 265 fungi, and 117 protozoa”; RVFV spiked into *Culex* mosquitoes were successfully detected by the platform (69).

#### BSL-2 Reagent Production

The select agent status of RVFV limits the tests and control material that can be handled by veterinary diagnostic laboratories that operate at BSL-2. Therefore, Drolet et al. “modified an existing one-step real-time RT-PCR (rRT-PCR) assay for quick virus inactivation and use in BSL-2 laboratories” (70). Further, an immunohistochemical (IHC) assay was developed for use by BSL-2 laboratories, using antiserum against recombinant RVFV-nucleocapsid (N) (70). These advancements provide necessary diagnostic tools to public health and veterinary labs to safely test samples for RVFV and thereby enhance capacity for rapid disease detection and response activities for this high containment pathogen.

#### Diagnostic Considerations

When considering a diagnosis of RVF in animals in the US, the symptoms observed tend to be non-specific. Therefore, the below diseases are recommended to comprise the testing panel for diagnosis: anthrax, bacterial septicemias, bluetongue, brucellosis, heartwater, ephemeral fever, enterotoxemia of sheep, Nairobi sheep disease, ovine enzootic abortion, peste des petits ruminants, toxic plants, trichomonosis, vibriosis, and Wesselsbron disease (40). Due to biosafety and biosecurity concerns, aerosolization potential, and select agent status, diagnostic testing for suspected RVF cases would be performed at the Foreign Animal Disease Diagnostic Laboratory (FADDL), part of the National Veterinary Services Laboratories (NVSL) (40).

## Japanese Encephalitis

Globally, Japanese encephalitis virus (JEV) is one of the most prevalent encephalitic arboviruses, mainly found in eastern and southern regions of Asia (71). Japanese encephalitis virus is primarily recognized as a human pathogen, but affects animal health as well. This virus is transmitted enzootically among mosquitoes (i.e., *Culex tritaeniorhynchus*), swine and wading birds, which serve as amplification hosts (72). Adult pigs are typically asymptomatic to JEV infection, however the virus is known to cause severe reproductive complications and fetal abnormalities among infected swine (41). Infection of horses with JEV has also been documented (73). People are exposed to JEV through the bite of an infectious mosquito but are considered dead-end hosts, as there is no human-to-human transmission. Japanese encephalitis virus is comprised of five genotypes (GI–GV) which are classified according to the phylogeny of the envelope (E) gene (74), with emergence and circulation of different genotypes alerting public health professionals throughout areas of endemicity. Commercial vaccines are available for humans (75), and pigs and horses (76), however veterinary vaccines for JEV are relatively less available than human vaccines (41).

### Surveillance

Japanese encephalitis virus (JEV), West Nile virus (WNV), and St. Louis encephalitis virus (SLE) share the same serocomplex (77). There is existing field and laboratory surveillance infrastructure for these other flaviviruses in the US. Operationally, collection and testing of *Culex* mosquitoes and sentinel surveillance programs in place for WNV could simultaneously also support vector surveillance for JEV through expansion of the testing panel, and funding should allow for supplementary surveillance activities.

The WHO publication, the “Manual for the Laboratory Diagnosis of Japanese Encephalitis Virus Infection” (43), covers JEV epidemiology, the role of the laboratory in JEV surveillance, prevention, and control, coordination of laboratory activities at local and national scales, specimen collection and handling, laboratory diagnosis procedures, testing algorithms/workflow, and data management (Table 2). Similarly, the WHO publication, “WHO-recommended standards for surveillance of selected vaccine-preventable diseases” (42) provides surveillance guidelines for a number of diseases including JE. Information included in this report include the accepted case definition, laboratory criteria for confirmation, and recommended types of surveillance (Table 2). Comprehensive syndromic surveillance for acute encephalitis syndrome (AES) is recommended for JEV (42). These resources will provide some initial guidance in establishing a public health laboratory surveillance network and testing algorithm for JEV in the US that is consistent with global recommendations.

Surveillance for JEV in the US would comprise passive and active surveillance of mosquito vectors, wildlife hosts, and domestic animals as well as people. Considering the genotype diversity of JEV, phylogenetic models have been developed to study JEV host changes and patterns of dispersal for different JEV genotypes using data collected through active and passive surveillance programs. Through this study, the authors concluded that active surveillance of mosquitoes was important in characterizing the circulating strain diversity of JEV, epidemiology, and transmission patterns, which informed virus spatial and evolutionary patterns (78). Yoshikawa et al. studied the serological and molecular epidemiology of JEV on an island of Japan, by conducting active surveillance of pigs as well as mosquitoes. Pigs were sampled at slaughterhouses and pig farms, and mosquito collections were coordinated near pig farms. The resulting data demonstrate seasonality of virus-positive mosquitoes and seroconversions and virus isolation from pigs over a 7-years period (19). This study presents the type of information that is useful in identifying not only spatial foci of transmission, but also seasonal patterns, which will direct evidence-based surveillance and control programs and help conserve resources. Vector surveillance would play a key role in monitoring JEV circulation after a virus introduction.

The practice of using of sentinel animals to detect active transmission through seroconversion is widespread in arbovirus surveillance programs. Cohorts of sentinel pigs in addition to mosquito collections have been used to determine the rate of JEV transmission to pigs in Cambodia (79). While virus infection rates in mosquitoes were low, intense transmission was detected in pigs through the seroconversion of almost all of the pigs over the course of the study. This finding may be attributed to the potential for transmission of JEV directly between pigs through oral-nasal secretions (80), providing further support for an integrated surveillance program involving vector and vertebrate sampling. Chickens have also served as sentinel animals for detecting virus circulation in serological surveillance programs for WNV, JEV (81), and other flaviviruses (82).

### Diagnostic Tools

A variety of serological and molecular diagnostics, using both single- and multiplex platforms, have been developed for detection of JEV (43).

#### Serological Diagnosis

Antibody detection (IgM and IgG) by capture ELISA is the recognized standard for JE diagnosis (83, 84). However, an important consideration for JE serological diagnosis is the extraordinary cross-reactivity among flaviviruses due to shared viral epitopes (85). This cross-reactivity challenges the ability of diagnosticians to verify the identity of the infecting flavivirus, particularly in endemic areas where multiple flaviviruses are co-circulating. Diagnostic approaches to overcome these challenges with flavivirus serology include the use of specific IgM ELISAs during the early stages of infection, and plaque reduction neutralization test (PRNT) (43). While PRNT may provide serological differentiation among closely-related and/or co-circulating flaviviruses, the technique is labor-intensive and involves working with live virus, which may not be feasible for many laboratories.

Currently, the recommended method for JE diagnosis of human infections is the JEV-specific IgM antibody-capture enzyme-linked immunosorbent assay (MAC-ELISA) in CSF and serum (86). Several commercial ELISA kits have been developed for the diagnosis of JEV and other flaviviruses. These kits offer a standardized testing method for widespread use, however sensitivity and specificity issues complicate interpretation of results and may mis-inform stakeholders due to false negatives or antigen-reactivity. The “JEV MAC-ELISA” has been used since 2006 for laboratory-based surveillance of JE by the WHO Japanese encephalitis (JE) laboratory network (86). Of the available MAC ELISA kits available, comparative testing determined that the “Panbio JE-Dengue IgM combo ELISA” (Inverness Medical Innovations Inc., Queensland, Australia) had a higher specificity than either the “InBios JE Detect™ MAC-ELISA (JE Detect)” (InBios International Inc., Seattle, WA) or the JEV CheX (XCyton Diagnostics Ltd., Bangalore, India) kits, however the PanBio kit has not been commercially produced since 2013 (86). Johnson et al. screened a panel of JEV+ and DENV+ control serum and CSF with JE Detect and DENV Detect InBios kits and developed a testing algorithm for differential diagnosis of these two viral infections (86).

Recognizing the problem of serological cross-reactivity among flaviviruses and its impact on surveillance and diagnosis, Cleton et al. used sera from naturally and experimentally-infected horses to develop a protein microarray to differentiate flaviviral infections in horses by NS1-antigen (87). Differentiation among flaviviral infections in horses using this method was possible, however some cross-reactivity was still noted, and differentiation between vaccinated and infected horses was not possible. Still, this type of platform could be a tool for screening serum samples of symptomatic individuals for multiple flaviviruses at once (87). Microseroneutralization tests have also been used in serosurveillance of JEV (88).

#### Molecular Detection

JEV viral RNA can be detected from viremic vertebrate samples and mosquitoes by many different available assays spanning traditional single-plex RT-PCR (89, 90), to more complex multiplex assays to screen samples for multiple arboviruses simultaneously (91). The multiplex assay developed by Wang et al. targets; “WNV, Saint Louis encephalitis virus, Venezuelan equine encephalomyelitis virus, Western equine encephalomyelitis virus, Eastern equine encephalomyelitis virus, Highlands J virus and JEV” (91).

## African Swine Fever

African swine fever virus (ASFV) (*Asfaviridae: Asfavirus*), is transmitted among wild vertebrate reservoir species by soft ticks in the genus *Ornithodoros*. Infection of wild and domestic suids with this virus is associated with high mortality, and hemorrhagic fever, however infections in African suid species are asymptomatic (92). Until the 1980s ASF remained confined to Africa, with the exception of Sardinia. In 2007 ASF was reported from the country of Georgia (93). From Georgia, ASFV expanded its range throughout the Caucasus and the Russian Federation, and was reported from the European Union (EU) in 2014. Incursions of ASFV into Caribbean and Brazil were met with aggressive and costly responses that successfully eradicated the virus (94). ASFV strains belonging to the vp72 genotype II currently circulate endemically among wild boar populations in Eastern Europe and cause epidemics in domestic pig and wild boar populations, while genotype I circulates in Sardinia. From this history, many lessons have been learned from curtailing ASFV circulation in resource-poor endemic areas in Africa, as well as combatting outbreak activity in areas of recent introduction throughout Europe.

### Surveillance

While ASFV is known to be transmitted by ticks in the genus *Ornithodoros*, most surveillance efforts are focused on disease detection and diagnosis in domestic and wild suids, perhaps due to the inconsistent involvement of ticks in outbreak situations. In eastern and southern African countries, ASFV circulates enzootically between ticks of the *O. moubata* species complex and common warthogs. In contrast, transmission in West Africa, appears to occur among domestic pigs in the absence of ticks (95). All available experimental data to date on which vertebrate species become viremic upon infection with ASFV is restricted to animals in the family Suidae, and was reviewed by Golnar et al. Of these hosts, domestic pigs produced the highest circulating virus titers, with bush pigs and warthogs producing lesser but detectable viremias (30). *Ornithodoros erraticus* was determined to be responsible maintaining ASFV transmission in outdoor pig production facilities on the Iberian Peninsula. This is in contrast to many other locations throughout Europe in which the distribution and role of *Ornithodoros* ticks in the transmission of ASF is poorly known (95). A recombinant rtTSGP1 ELISA kit can detect antibodies to *O. moubata* salivary gland proteins with 100% sensitivity and 99.4% specificity and directly determines the exposure history of pigs to blood feeding ticks (96). This and similar tests can therefore be used in addition to conventional tests for surveillance to determine potential exposure that could portend risk to ASF through ticks. While it will be important to understand the competence and distribution of potential tick vectors of ASFV in the US and areas at risk of ASFV establishment (30, 32), efforts to monitor for an introduction event should likely focus on evidence for infection among wild and domestic suids. Once ASFV establishes itself in a new area, the role of ticks will become increasingly more important to the environmental maintenance of this virus.

Passive surveillance of deceased wild boar and symptomatic animals could serve as first indicators of ASFV activity in new area of introduction (97, 98). Petrov et al. demonstrated that molecular detection of ASFV as well as classical swine fever virus were also detected reliably by quantitative PCR from dry and semi-dry blood swabs collected from carcasses (98). Similarly, Carlson et al. evaluated the reliability of detecting ASFV antibody from blood swabs taken from deceased animals in the field using commercially-available antibody-based detection kits (97). Sensitivity and specificity of these kits on dried blood swabs well-exceeded 90%. These studies demonstrate that molecular and serological diagnostics are easily applied to a passive surveillance strategy targeting carcasses, and a practical approach for early detection of ASFV circulation.

Mur et al. tested oral fluids as a non-invasive alternative to serum for ASF diagnosis (99). Oral fluid samples were collected by allowing experimentally-infected pigs to chew on ropes, and collecting fluid from the ropes. Paired samples of oral secretions and serum were tested for antibody against ASFV by both ELISA and the immunoperoxidase test (IPT). ASFV antibodies were detected in oral secretions for the 65-days duration of the experiment by both methods, introducing the possibility of saliva-based surveillance and diagnostics for ASF.

Fernandez-Carrion et al. developed a motion-based video surveillance system, based on the concept of monitoring animal behavior for signs of illness characteristic ASFV disease (100). Among experimentally-infected pigs, changes in mobility patterns preceded the onset of other disease symptoms (100). Further, motion-based video surveillance can be combined with temperature monitoring via biosensors, and linked to a smart system to inform care takers when animals become feverish and/or lethargic (101). This system offers a non-invasive early-warning system for commercial animal facilities.

One challenge to surveillance is the potential for some suids to serve as asymptomatic carriers of ASFV and drive epizootic activity. Pigs surviving infection can contribute to virus spread within the population as persistently-infected carriers (102). Abworo et al. studied infection of pigs with ASFV genotype IX in small farming operations along the border of Kenya and Uganda (103). Approximately 16% of clinically-healthy pigs sampled had ASFV detected in their tissues, suggesting a role for persistently-infected pigs maintaining the virus in these production systems (103). Bellini et al. reviewed precautionary measures focused on minimizing the risk of spreading ASFV in and among pig farms in the epidemiological context of European systems (104).

Several gaps and priorities have been identified relative to ASFV control and surveillance: “(1) raise awareness among hunters, farmers and veterinarians, (2) ensure farm locations are far from suitable wild boar areas, and in affected areas, promote confinement, (3) prepare early warning systems, contingency plans, and control measures, (4) implementation of surveillance activities based on the risk of potential exposure, introduction, and spread” (105). Strict sanitation and early detection of ASFV in new areas are paramount, because no vaccine is currently available (102). Educational campaigns will be instrumental in raising awareness among key stakeholders to ensure rapid detection and response (106). Rapid disease recognition in the field and on farms is critical, followed by laboratory confirmation. Awareness of hunters, farmers, and veterinarians to the signs and symptoms of ASF, further understanding of ASFV epidemiology in areas of introduction, and preparedness with appropriate diagnostic capabilities are all critical next steps to the detection and mitigation of an introduction of ASFV into the US. The USDA-APHIS Veterinary Services (VS) recently proposed an integrated active surveillance plan for ASF and classical swine fever targeting higher-risk populations, sick pigs, and mortality events. Currently, detection of ASF will rely on passive disease reporting. The approval of tonsil and spleen tissues as additional valid tissue types for diagnostic testing in the National Animal Health Laboratory Network (NAHLN) presents an opportunity to integrate ASF surveillance with current CSF surveillance efforts as an active surveillance plan for swine diseases (46). USDA APHIS has also posted a disease response strategy for ASF (45). A subset of NAHLN laboratories has trained and proficiency tested analysts ready to participate in foreign animal disease investigations or surge capacity testing as needed (107).

### Diagnostic Tools

Multiple molecular and serological detection platforms exist for ASFV, and are reviewed in detail by Gallardo et al. (102) and Arias et al. (105). Importantly, Gallardo et al. also provide valuable information on the interpretation of ASF diagnostic results in the context of differing clinical presentation and strain diversity (108).

#### Serological Detection

Currently, there is no approved vaccine for ASFV. Therefore, all antibody detections in field samples are indicative of natural exposure to virus circulation. Though caution must be taken in endemic epidemiological situations zero antibody positivity has been observed PCR ASF positive animal (109). Average animal positivity for ASFV by PCR in Southwestern Kenya was 28%, despite these animals being asymptomatic and sero-negative by ELISA (109). Such results demand for parallel confirmatory test to rule out infection or exposure, e.g., combination of molecular tools and serology. Gallardo et al. compared three commercial ELISAs with varying antigenic targets, the OIE-ELISA test, and the confirmatory immunoperoxidase test (IPT) (102). While the ELISA-based tests were rapid, high-throughput, and could be automated, sensitivity was generally poor compared to that of the Universal Probe Library (UPL-PCR) molecular test (see below) (102). The IPT was advantageous in detection of ASF antibodies earlier during the course of infection when titers were low (102). More recently, an indirect ELISA can be used to detect ASFV antibodies in either serum or oral secretions, and has been validated on field samples (110). Serological assays optimized for use on both oral and serum samples will be useful in endemic areas where virulence is low (110).

#### Molecular Detection

Recommended PCR assays for ASFV have become standard diagnostic tool in reference laboratories (111, 112). A side-by-side proficiency test of the UPL-PCR (113), and the recommended OIE conventional (44, 111) and real-time PCR assays (44, 111) assays revealed almost perfect agreement between these methods, however the UPL-PCR had greater diagnostic sensitivity for early detection of the disease. Due to some potential primer mis-matches between the primers in the OIE PCR assay (111). Luo et al. designed novel conventional PCR primers to address these sensitivity issues observed with the OIE assay for detection of European strains of ASFV (114). This updated PCR assay demonstrated improved diagnostic sensitivity than the two OIE over comparable PCR assays (111, 112) when detecting diverse virus strains (114). Wozniakowski et al. recently developed a polymerase cross-linking spiral reaction (PCLSR). This sensitive new test does not cross-react with other porcine pathogens, and has been validated on multiple sample types derived from ASFV-infected wild boars and pigs (115).

Because ASF is only one porcine pathogen of veterinary significance, some diagnostic efforts have included ASF in multiplex assays targeting multiple agents. Grau et al. developed a multiplex real-time PCR (mRT-qPCR) for concurrent detection of ASFV, classical swine fever virus, and foot and mouth disease virus, and evaluated use of this assay on swine oral secretions (116). This mRT-qPCR consisted of previously published single-plex RT-qPCR or qPCR assays (117–119) in use at the USDA-NVSL-FADDL and within the NAHLN, that for this study were combined into the multiplex format (116). A multi-plexed real time PCR assay has also been developed for ASFV and classical swine fever virus (120).

## Discussion

While all three of these pathogens are arthropod-borne viruses, the unique biology of these three systems demonstrates that there is no single approach for the surveillance and diagnostics. Vector, animal, and public health infrastructure needs to sustain broad capacity to rapidly detect and respond to these three viruses and more. The NVSL system, including the over 60 NAHNL labs and the FADDL will play a critical role in the diagnostic testing and confirmation of these disease agents. Currently, many NAHLN laboratories are already approved for ASFV testing (107). While vector collection and vector-based surveillance approaches are an integral component to post-introductory surveillance and management (Table 2), the first indicators of an introduction event of any of these diseases would likely be through recognition of sick wildlife or domestic animals or pathogen detection from carcasses. The USDA foreign animal diseases preparedness plans for each of these transboundary viruses includes a component of animal culling for biocontainment purposes, together with vector control and vaccination, if possible (40, 45) (Table 2). With this in mind, capacity for passive surveillance focused on the recognition of disease symptoms in domestic and wild animals by appropriate professionals (i.e., producers, veterinarians, wildlife professionals), particularly those near high risk ports of entry and in first-responding agencies, should be prioritized in order to detect and diagnose these cases early. Rapid response and containment will minimize the spread of the disease, and broader impacts on producers. Aspects of these diseases that have been exploited as predictors of virus transmission in other parts of the world may also be effective surveillance tools in the US, including outbreak forecasting using climate models for RVF. Vector surveillance activities will likely be most informative and important immediately after initial pathogen detection in animals or humans. Vector surveillance will help to incriminate particular vector species, determine vector abundance and infection rates, genotype circulating virus strains, and evaluate the efficacy of control strategies on vector populations and pathogen infection rates. National or international guidelines on surveillance and diagnosis also exist for each of these three agents (Table 2). These documents comprise a valuable starting place for critical discussions and development of action plans for surveillance and response activities in the US.

## Author Contributions

GH and RK: conceptualization. GH: funding acquisition. RK: original draft. RK, GH, and EA: review and editing.

### Conflict of Interest

The authors declare that the research was conducted in the absence of any commercial or financial relationships that could be construed as a potential conflict of interest.
